# MiR-124 acts as a tumor suppressor by inhibiting the expression of sphingosine kinase 1 and its downstream signaling in head and neck squamous cell carcinoma

**DOI:** 10.18632/oncotarget.15334

**Published:** 2017-02-15

**Authors:** Yuan Zhao, Zhiqiang Ling, Yubin Hao, Xiaowu Pang, Xianlin Han, Joseph A. Califano, Liang Shan, Xinbin Gu

**Affiliations:** ^1^ Department of Oral Pathology, College of Dentistry, Howard University, Washington DC, USA; ^2^ Zhejiang Cancer Hospital, Zhejiang Cancer Research Institute, Hangzhou, Zhejiang, China; ^3^ Sanford Burnham Prebys Medical Discovery Institute, Orlando, Florida, USA; ^4^ Department of Otolaryngology, Head and Neck Surgery, Johns Hopkins University, San Diego, California, USA; ^5^ Department of Radiology, College of Medicine, Howard University, Washington DC, USA; ^6^ Cancer Center, Howard University, Washington DC, USA

**Keywords:** miR-124, sphingosine kinase 1, ceramide, BcL-2 family, head and neck cancer

## Abstract

By analyzing the expression profile of microRNAs in head and neck squamous cell carcinomas (HNSCC), we found that the expression level of miR-124 was 4.59-fold lower in tumors than in normal tissues. To understand its functions, we generated a miR-124-expressing subline (JHU-22^miR124^) and a mock vector-transfected subline (JHU-22^vec^) by transfecting the mimic of miR-124 into JHU-22 cancer cells. Restored expression of miR-124 in JHU-22^miR124^ cells led to reduced cell proliferation, delayed colony formation, and decreased tumor growth, indicating a tumor-suppressive effect of miR-124. Subsequent target search revealed that the 3′-UTR of SphK1 mRNA carries a complementary site for the seed region of miR-124. SphK1 was also detected to be overexpressed in HNSCC cell lines, but down-expressed in JHU-22^miR124^ cells and tumor xenografts. These results suggest that SphK1 is a target of miR-124. To confirm this finding, we constructed a 3′-UTR-Luc-SphK1 vector and a binding site-mutated luciferase reporter vector. Co-transfection of 3′-UTR-Luc-SphK1 with miR-124 expression vector exhibited a 9-fold decrease in luciferase activity compared with mutated vector, suggesting that miR-124 inhibits SphK1 activity directly. Further studies on downstream signaling demonstrated accumulation of ceramide, increased expression of the pro-apoptotic Bax, BAD and PARP, decreased expression of the anti-apoptotic Bcl-2 and Bcl-xL, and enhanced expression of cytochrome c and caspase proteins in JHU-22^miR124^ compared with JHU-22^vec^ cells and tumor xenografts. We conclude that miR-124 acts as a tumor suppressor in HNSCC by directly inhibiting SphK1 activity and its downstream signals.

## INTRODUCTION

MicroRNAs (miRNAs) are a group of small non-coding RNA molecules that function in RNA silencing and post-transcriptional regulation of gene expression *via* base-pairing with the complementary sequences within mRNA molecules. Since the discovery of miRNAs in 1993, great efforts have been made on the expression profiles and functions of miRNAs in different tissues and biological processes [1, 2]. One significant achievement is the finding that miRNAs are expressed and function aberrantly in various types of cancer [3–6]. It is predicted that miRNAs regulate over 30% of protein-coding genes that are closely associated with cancer development and progress [7–9]. Because of these findings, miRNAs have been extensively explored as biomarkers for cancer diagnosis and prognosis prediction. MiRNA-based therapies are also under investigation [10, 11].

In recent years, studies have also been conducted on the expression profile of miRNAs in head and neck squamous cell carcinoma (HNSCC) [12–15]. A group of miRNAs, including miR-21, miR-155, miR-31 and miR-223, has been consistently shown to be up-expressed, while another group of miRNAs, including miR-375, miR-1, miR-133a, miR-99a, miR-125b, miR-100, miR-143 and miR-204, has been shown to be down-expressed in HNSCC [12–15]. However, there is considerable variability in the expression of many miRNAs among reports. For example, a certain group of miRNAs exhibits a complicated expression pattern that varies among different cell types and tumor tissues, as well as at different stages of tumor progression. Several mechanisms have been suggested for the altered expression of miRNAs, including direct genetic loss, alterations in their biogenesis pathway, epigenetic changes, altered transcription factor expression, and changes to their target site [16]. However, whether the altered miRNA expression patterns are the direct cause of cancer or are an indirect effect of changes in cellular phenotype remains to be answered. It is also notable that a single miRNA can regulate multiple targets [17]. Consequently, it can be difficult to classify a miRNA as an oncogene or a tumor suppressor [12].

To identify the miRNAs with aberrant functions in HNSCC, thus developing novel diagnostic and therapeutic approaches, we analyzed the expression profile of a set of cell proliferation-associated miRNAs in human HNSCC. Of them, miR-124 showed significantly reduced expression in HNSCC compared with normal tissues. This finding prompted investigation into whether miR-124 is involved in HNSCC, what function it plays, and what the downstream signaling is underlying its function. Literature review has shown that miR-124 is a miRNA that is still controversial in its expression and function in cancer. MiR-124 is a highly conserved miRNA. Its mature sequence is processed from three precursor variants that are located at chromosomes 8p23.1 (miR-124-1), 8q12.3 (miR-124-2) and 20q13.33 (miR-124-3), respectively. Studies have shown that miR-124 is down-expressed in various types of cancer, which is inversely associated with tumor growth, lymph node metastasis, and poor prognosis [18–22]. However, there are studies that show different expression patterns and functions of miR-124 in cancer. For example, Eslahi *et al*. reported that miR-124 is overexpressed in lymph node metastasis of breast cancer compared with primary tumors [23]. Chen *et al*. have shown that increased miR-124 expression correlates with better breast cancer prognosis, specifically in patients receiving chemotherapy [24]. The expression and role of miR-124 in HNSCC is still poorly understood and the molecular mechanisms by which miR-124 acts on tumor cells remain largely unknown. In the present study, we found that miR-124 acted as a tumor suppressor in HNSCC *via* directly inhibiting the expression of sphingosine kinase 1 (SphK1), a core enzyme that regulates the ceramide-sphingosine-sphingosine-1-phosphate (S1P) interconversion, ultimately directing cells towards an apoptotic program in HNSCC.

## RESULTS

### Identification of miR-124 as an aberrantly expressed miRNA in HNSCC tumors and cell lines

**Figure 1:**
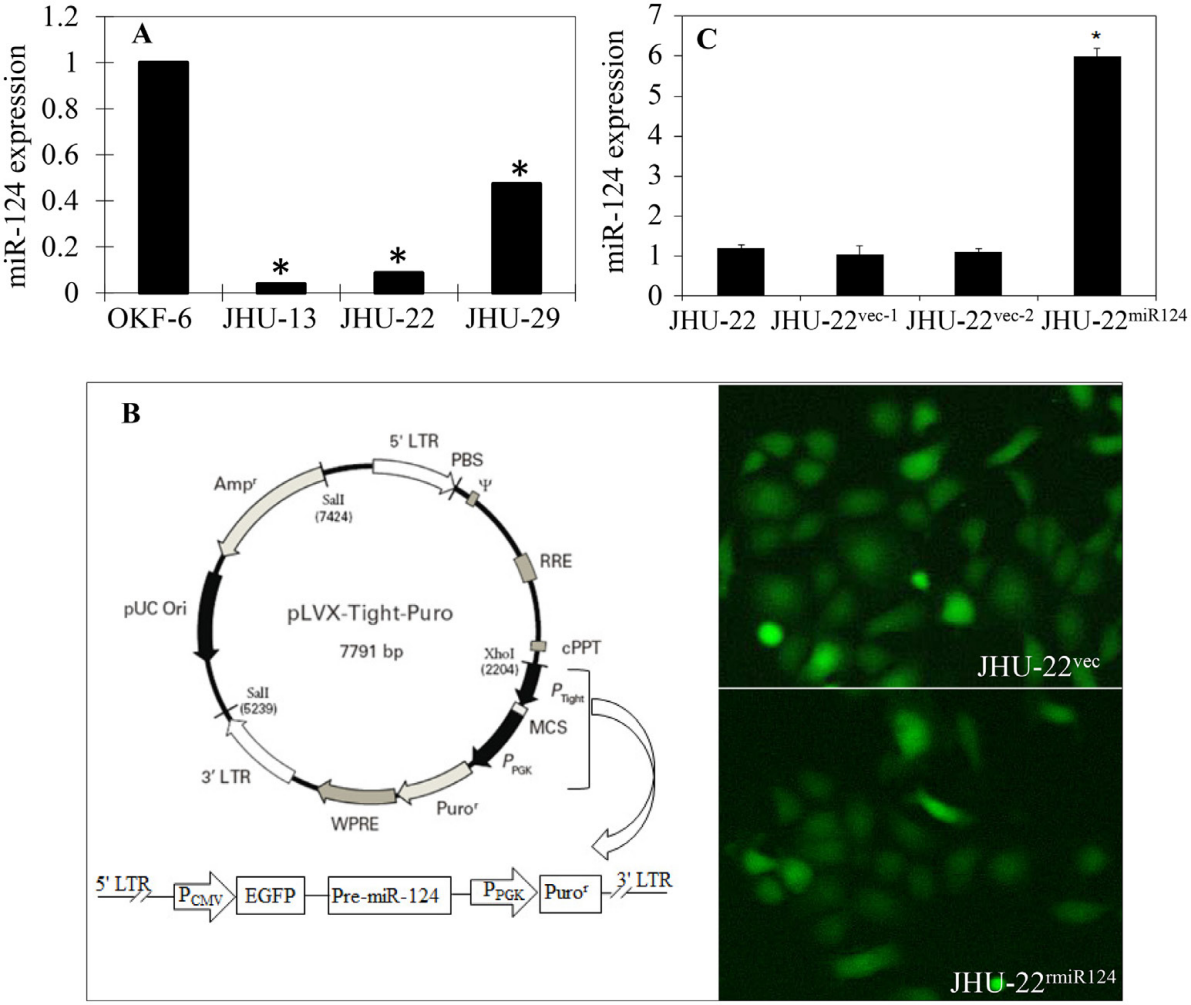
Decreased expression of miR-124 and restoration of its expression in HNSCC cell lines **A**. Decreased expression of miR-124 in three HNSCC cell lines (JHU-13, JHU-22 and JHU-19), compared to the immortalized keratinocyte line OKF-6 by QRT-PCR (*P<0.05 for all three cancer lines). **B**. The construct of the EGFP-miR-124 lentivirus expression vector (left panel) and the fluorescent images of JHU-22^vec^ and JHU-22^miR124^ cells after transfection (right panel). The P_tight_ promoter in the commercial pLVX-Tight-Puro vector was replaced with an expression cassette containing a full CMV promoter (P_CMV_), enhanced green fluorescent protein (EGFP), and pre-miR-124, and linking to a selective cassette containing the P_PGK_ promoter and Puro^r^. The pre-miR-124 sequence was removed in the mock vector. The lentivirus particles were produced in the HEK293T packaging cells and introduced into the JHU-22 cancer cells. The fluorescence from EGFP expression in the JHU-22^vec^ and JHU-22^miR124^ transfected cells indicated successful transfection of the plasmid vectors into the JHU-22 cell line. **C**. Establishment of cell lines with stable expression of miR-124, showing six-fold higher for the expression of miR-124 in JHU-22^miR124^ cells than in mock transfected JHU-22^vec^ cells and the parental JHU-22 cells with QRT-PCR (*P<0.05).

To identify the miRNAs with aberrant functions in HNSCC, we first analyzed the expression profile of a set of 12 cell proliferation-associated miRNAs in HNSCC tumors and tumor-adjacent normal tissue samples using quantitative reverse transcription PCR (QRT-PCR) [5, 6]. Of them, four miRNAs, including miR-21, miR-200a, miR-200b, and miR-429, exhibited markedly increased expression. Only miR-124 showed significantly reduced expression (4.59-fold decrease) in HNSCC tumors compared with tumor-adjacent normal tissues (*P*<0.05, Table 1). Accordingly, we focused studies on the function of miR-124 in HNSCC. We first analyzed the expression of miR-124 in three HNSCC cell lines, including JHU-13, JHU-22 and JHU-29, and in a noncancerous oral keratinocyte cell line OKF-6. JHU-13 cell line was established from neck lymph node metastasis, and JHU-22 and JHU-29 cell lines were established from primary tumors of the larynx and the tongue base, respectively. QRT-PCR revealed that miR-124 was also significantly down-expressed in all three HNSCC cell lines compared with OKF-6 line (*P*<0.05, Figure 1A). These results suggest that miR-124 may function abnormally in HNSCC.

**Table 1:** The expression profile of cell growth-associated miRNAs in human HNSCC tumors

miRNAs	Fold change*	*P*-value
miR-21	8.07	0.009**
miR-101	1.44	0.567
miR-125b	-2.13	0.050
miR-145	2.50	0.135
miR-34a	2.55	0.164
miR-141	4.98	0.063
miR-200a	6.95	0.020**
miR-200b	5.39	0.047**
miR-200c	2.13	0.329
miR-429	5.70	0.031**
**miR-124**	**-4.59**	0.036**
miR-218	4.08	0.050

*The expression in tumor to that in normal tissue; **P<0.05.

### MiR-124 acts as a tumor suppressor in HNSCC

#### Restored miR-124 expression inhibited HNSCC cell proliferation and colony formation

To understand the function of miR-124 in HNSCC, we constructed a miR-124 expression vector and transfected the miR-124 into JHU-22 cells to restore its expression. JHU-22 cell line was selected for the studies because JHU-13 is highly resistant to puromycin, which deems it unsuitable for selection process with puromycin. Figure 1B shows the construct of the EGFP-miR-124 lentivirus expression vector and its stable expression in transfected cells. This lentivirus vector contained an expression cassette with a P_CMV_ promoter, the enhanced green fluorescent protein (EGFP) sequence and the miR-124 precursor, and a selective cassette with a P_PGK_ promoter and the Puro^r^. EGFP expression in the transfected JHU-22 cells was used to monitor the transfection process (Figure 1B). The stably expressed clones for miR-124 and control were selected with puromycin. The cell clone with the highest expression of miR-124, designated as JHU-22^miR124^, was used for further studies, demonstrating six-fold higher expression of miR-124 than that in mock vector-transfected JHU-22^vec^ cells (Figure 1C).

**Figure 2:**
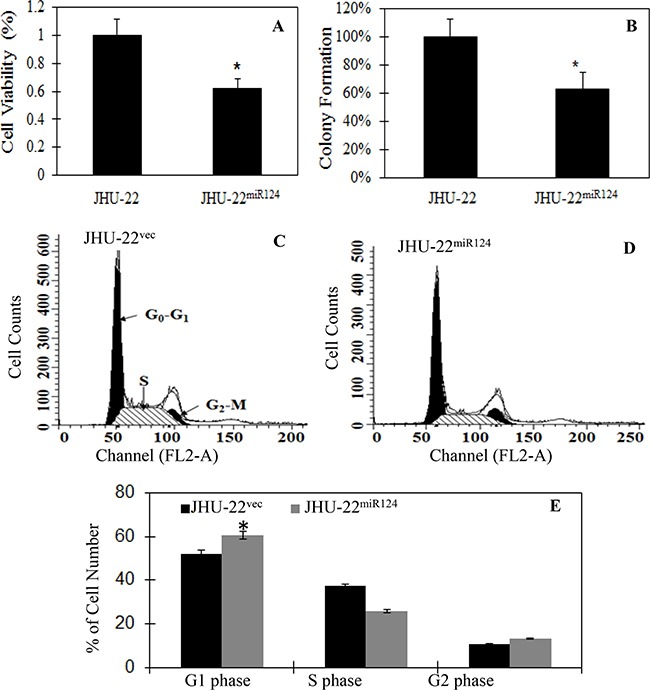
The effects of restored miR-124 expression on cell proliferation, colony formation and cell cycle distribution, showing inhibition of the tumor cell proliferation by MTT assay **A**., significant delay in colony formation with smaller and fewer colonies by colony formation assay **B**., and cell arrest in the G1 phase **C-E**. in JHU-22^miR124^ cells, compared with that in JHU-22^vec^ cells. The results represent the mean ± SD of two independent experiments with triplicates. * indicates *P*<0.05 between JHU-22^miR124^ and JHU-22^vec^ cells.

Following establishment of stably miR-124-expressing cells, we studied the effects of restored miR-124 expression on tumor cell proliferation, colony formation, and cell cycle distribution. Compared to JHU-22^vec^ cells, JHU-22^miR124^ cells demonstrated a 38% decrease in cell proliferation by MTT assay (Figure 2A), a significant delay in colony formation with smaller and fewer colonies (Figure 2B) by colony formation assay, and cell arrest in the G1 phase by flow cytometry (Figures 2C-2E). The cell doubling time was 21.87 ± 3.12 hours for JHU-22^vec^ and 24.49 ± 3.21 hours for JHU-22^miR124^ cells (*P*<0.05). These results suggest that miR-124 expression inhibits the HNSCC cell growth *in vitro*.

#### Restored miR-124 expression inhibited the growth of tumor xenografts

**Figure 3:**
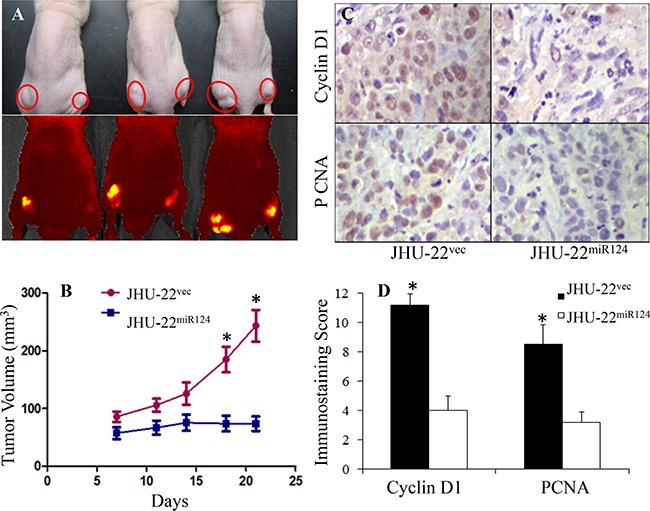
The effects of restored miR-124 expression on tumor xenograft growth, showing significant inhibition of tumor growth (*P*<0.05) **A**. Optical and fluorescent (EGFP as the reporter) images of three representative mice with JHU-22^miR124^ tumors in the right side and JHU-22^vec^ tumors on the left side of the mice (the circles indicate the tumors). **B**. The tumor volume change following tumor cell inoculation. **C and D**. Cyclin D1 and PCNA immunostaining showing decreased expression in JHU-22^miR124^, compared with that in JHU-22^vec^ tumor xenografts. * indicates *P*<0.05 between JHU-22^miR124^ and JHU-22^vec^ cells.

Considering that *in vitro* environments differ from *in vivo*, we further evaluated the effects of miR-124 expression on tumor growth in athymic nude mice. We inoculated both JHU-22^vec^ and JHU-22^miR124^ cells into the lower back subcutaneous tissue of the same mice for tumor growth comparison. When the mice were euthanized four weeks after tumor cell inoculation, solid tumor xenografts were developed in all mice (Figure 3A). Figure 3B shows the time-dependent volume changes of JHU-22^miR124^ and JHU-22^vec^ tumor xenografts (mean ± SD). The volume of JHU-22^miR124^ tumors was significantly smaller than that of JHU-22^vec^ tumors (*P*<0.05). At the end of experiment on day 28, the average weight of JHU-22^miR124^ tumors was only 30 mg, significantly lower than that of JHU-22^vec^ tumors (97 mg) (*P*<0.05).

Since the proliferating cell nuclear antigen (PCNA) and cyclin D1 represent two biomarkers of tumor growth, we also evaluated their expression in tumor xenografts. Immunohistochemistry showed that the expression of both PCNA and cyclin D1 were at least 50% lower in the JHU-22^miR124^ tumors, compared to their expression in the JHU-22^vec^ tumors from the same nude mice (*P*<0.05) (Figure 3C and 3D). All these results exhibited that restored miR-124 expression inhibited the growth of HNSCC cells and tumors, suggesting that miR-124 acted as a tumor suppressor in HNSCC.

### SphK1 was a direct target of miR-124 in HNSCC

#### SphK1 was highly expressed in HNSCC cells and its expression was inhibited by miR-124

**Figure 4:**
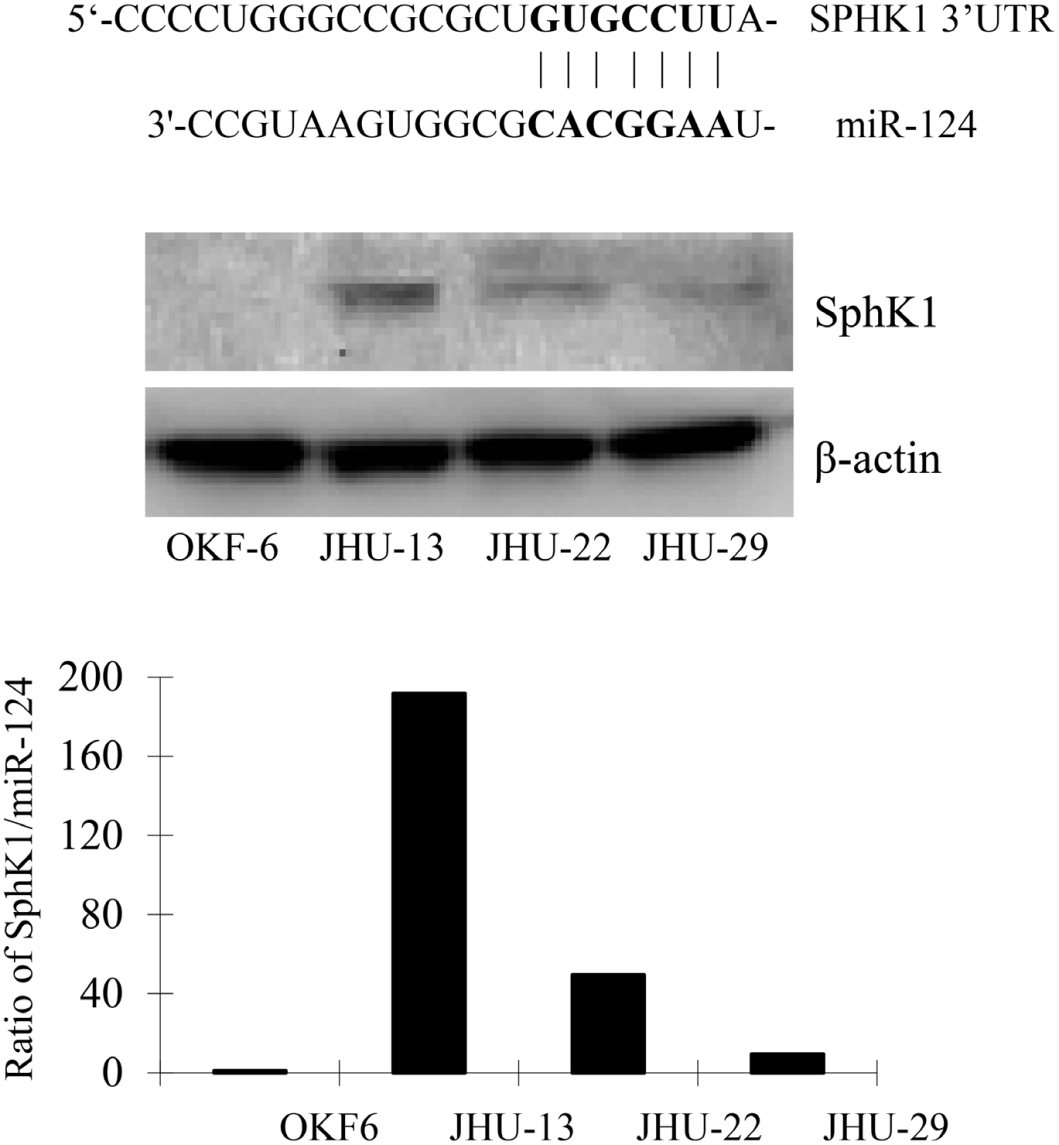
Upper panel shows the sequence of the 3′-UTR of SphK1 mRNA with a complementary site (3′-UTR 16-22) for the seed region of miR-124. Middle panel of Western blotting demonstrates higher expression level of the SphK1 in HNSCC cell lines compared with noncancerous OKF-6 cells. The lower panel shows the ratio of SphK1 to miR-124 expression in the cell lines with the highest in JHU-13 cells, followed by in JHU-22.

To understand the targets and tumor suppressive mechanisms of miR-124, we conducted a comprehensive search using the TargetScanHuman 7.0 and PicTar programs. We noticed that the 3′-UTR sequence of SphK1 mRNA carries a complementary site (3′-UTR 16-22) for the seed region of miR-124, suggesting SphK1 is a target of miR-124 (Figure 4, upper panel).

**Figure 5:**
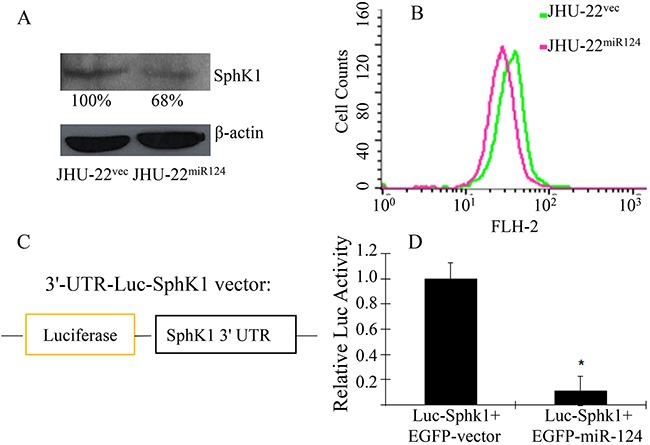
Inhibition of the SphK1 expression by miR-124 **A** and **B**. Decreased expression of SphK1 in JHU-22^miR124^ cells, compared with that in JHU-22^vec^ cells by Western blotting (5A) and flow cytometry (5B). The level of SphK1 in JHU-22^miR124^ cells was only 68% of that in JHU-22^vec^ cells. **C**. shows the construct of the 3′-UTR-Luc-SphK1 vector with a luciferase reporter and **D**. shows the luciferase activity after transfection into the HEK293T cells. At 48 h after transfection, the luciferase activity was 9-fold lower in cells co-transfected with 3′-UTR-Luc-SphK1 and EGFP-miR-124 vectors, than in cells co-transfected with 3′-UTR-Luc-SphK1 and mock EGFP-vectors (*P<0.05).

We then analyzed SphK1 protein expression in HNSCC cell lines. Western blotting revealed that the expression level of SphK1 was significantly higher in all three of the HNSCC cell lines, compared to its level in the noncancerous line OKF-6 (*P*<0.05 for all three lines). The JHU-13 cells had the highest level, followed by JHU-22 and JHU-29 cells. Compared to the miR-124 level, the highest ratio of SphK1/miR-124 expression was observed in JHU-13, followed by in JHU-22 and JHU-29 (Figure 4, middle and lower panels). There was a negative relationship between the expression levels of SphK1 and miR-124 in the three cancer cell lines. Upon restoration of miR-124 expression, SphK1 expression decreased; western blotting and flow cytometry showed that SphK1 level in JHU^miR124^ cells was only 68% of its level in JHU-22^vec^ cells (Figure 5A and 5B). These results suggest that SphK1 is a target of miR-124 and that miR-124 inhibits SphK1 expression in HNSCC.

#### MiR-124 directly regulated SphK1 expression

To investigate whether miR-124 directly regulates SphK1 expression, we constructed a 3′-UTR-Luc-SphK1 vector and a mutated 3′-UTR-Luc-SphK1 vector with luciferase activity as the SphK1 expression reporter. The former contained a fragment of the 3′-UTR SphK1 mRNA that carried the miR-124 complementary binding site (position 16-22 of SphK1 3′-UTR); the latter contained a mutated 3′-UTR SphK1 mRNA that changed the miR-124 binding site (Figure 5C). The 3′-UTR-Luc-SphK1 vector or the mutated 3′-UTR-Luc-SphK1 vector was co-transfected into HEK293T cells with either the EGFP-miR-124 expression vector or the mock EGFR vector without the miR-124 sequence. At 48 h after transfection, luciferase activity was 9-fold lower in HEK293T cells co-transfected with 3′-UTR-Luc-SphK1 and EGFP-miR-124 vectors, than in cells co-transfected with 3′-UTR-Luc-SphK1 and mock EGFP vectors (Figure 5D). In contrast, there was no significant difference in luciferase activity between HEK293T cells co-transfected with mutated 3′-UTR-Luc-SphK1 and EGFP-miR-124 vectors, and in cells transfected with 3′-UTR-Luc-SphK1 vector alone. These results suggest that miR-124 specifically binds with the 3′-UTR of SphK1 mRNA, thus inhibiting SphK1 expression.

### MiR-124-mediated inhibition of SphK1 expression led to ceramide accumulation in HNSCC tumor xenografts

Ceramides and S1P are two key signaling molecules that regulate cell fate decision, while SphK1 is a crucial enzyme in regulation of the ceramide-S1P balance [25–27]. Therefore, we analyzed whether miR-124-mediated inhibition of SphK1 expression could shift the ceramide-S1P balance toward ceramide in culture cells and tumor xenografts.

**Figure 6:**
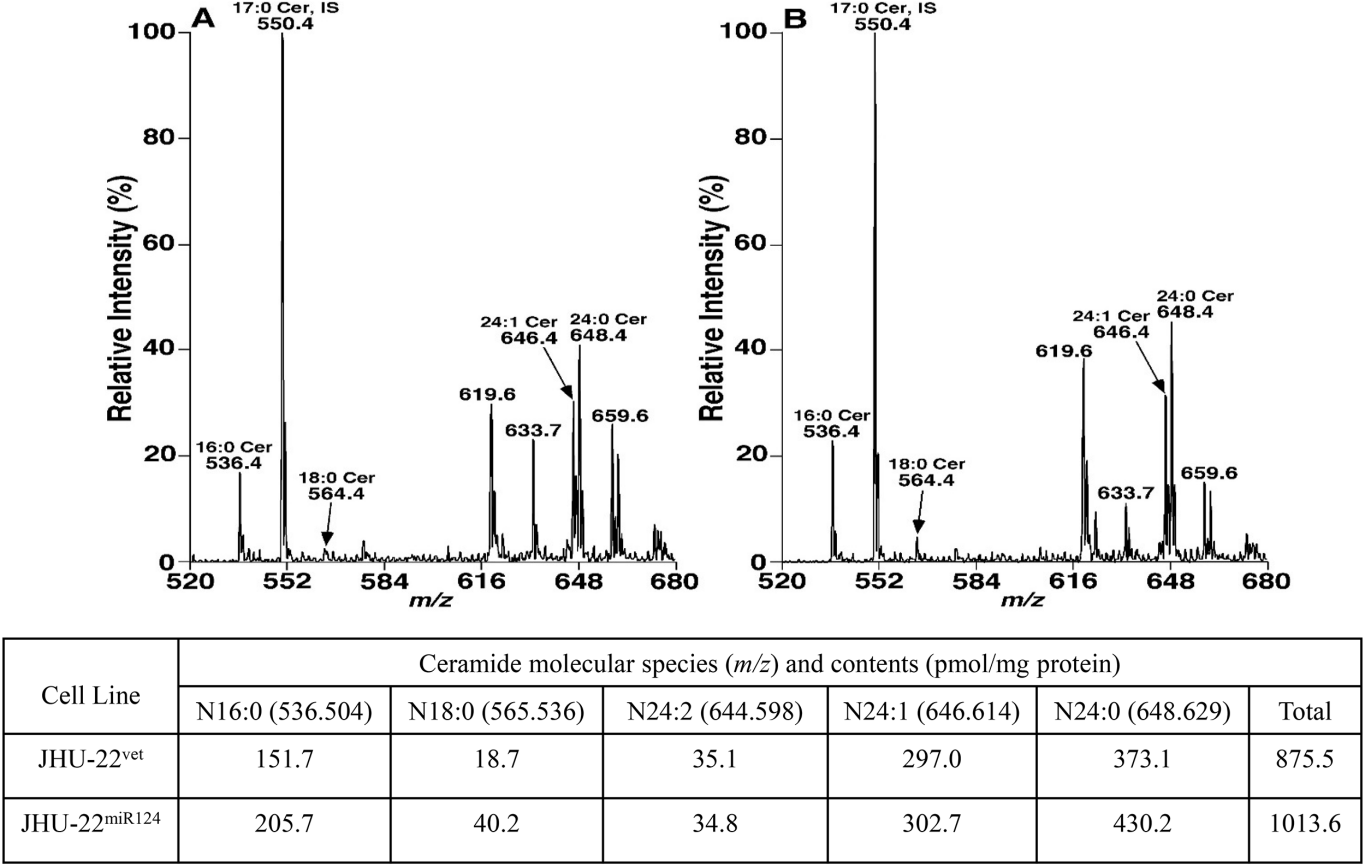
Mass spectra and quantification of ceramide molecular species **A** and **B**. are the spectra of species in the JHU-22^vec^ and JHU-22^miR124^ cells, respectively. The content of each ceramide molecular specie in the JHU-22^vec^ and JHU-22^miR124^ cells is presented in the table. The total ceramide content is 875.5 and 1013.6 pmol/mg protein in the JHU-22^vec^ and JHU-22^miR124^ cells, respectively, and is significantly different between them (*P*<0.05).

**Figure 7:**
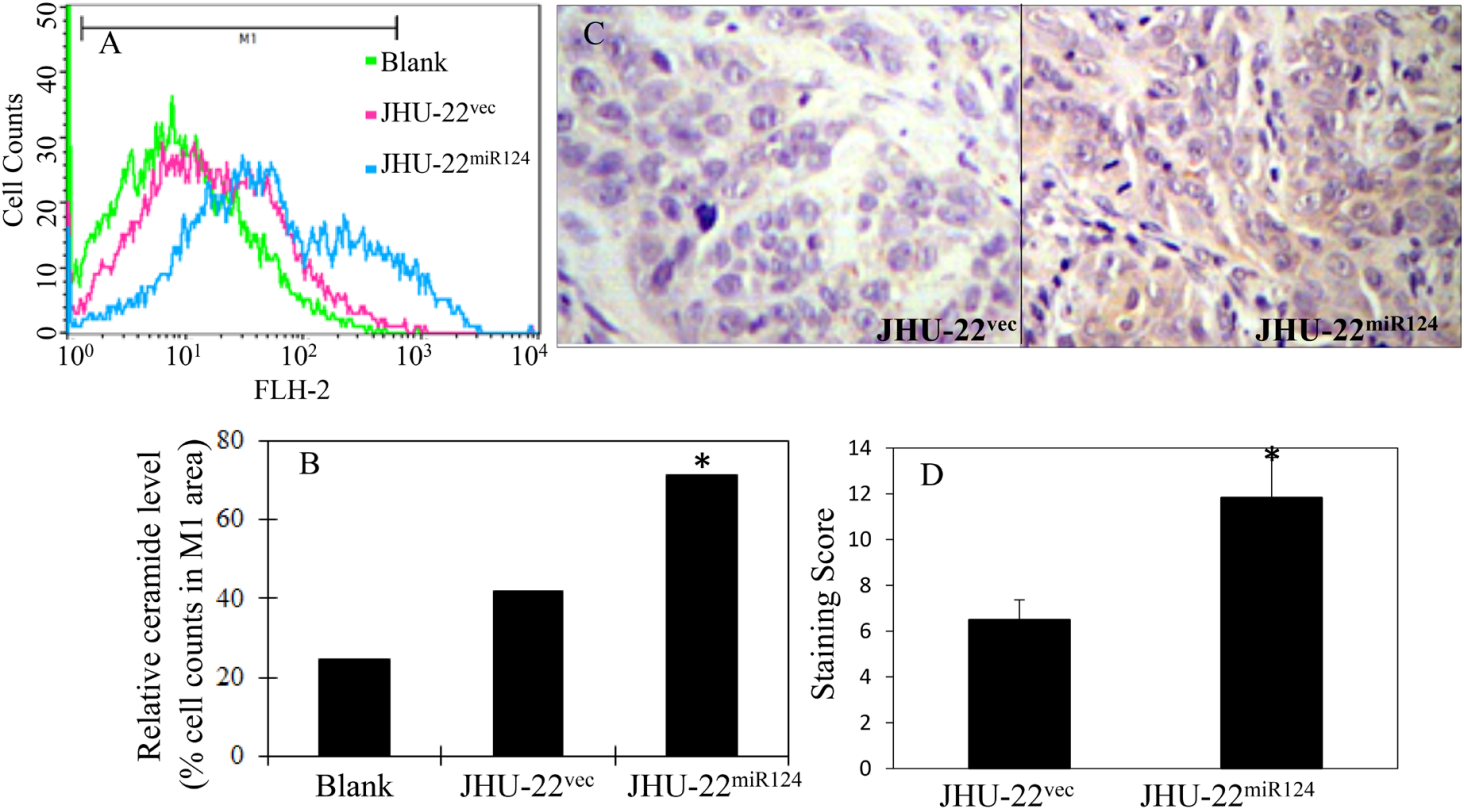
Flow cytometry and immunohistochemistry of ceramide level in the culture cells and tumor xenografts **A** and **B**. Flow cytometry demonstrated approximately 40% higher ceramide level in JHU-22^miR124^ than in JHU-22^vec^ cells (*P<0.05). **C** and **D**. Immunohistochemistry showed a stronger immunoreactive signal of ceramide in JHU-22^miR124^ than in JHU-22^vec^ tumor xenografts (*P<0.05).

We first quantified the levels of ceramides in JHU-22^vec^ and JHU-22^miR124^ cells using mass spectrometry. The total expression level of ceramides in JHU-22^miR124^ cells was 1013.6 pmol/mg protein, while in JHU-22^vec^ cells was 875.5 pmol/mg protein (*P*<0.05) (Figure 6). This difference was confirmed with flow cytometry and immunohistochemistry. Flow cytometry demonstrated approximately 40% higher ceramide level in JHU-22^miR124^ than in JHU-22^vec^ cells (Figure 7). Immunohistochemistry showed a stronger immunoreactive signal of ceramide in JHU-22^miR124^ than in JHU-22^vec^ tumor xenografts (Figure 7). All of these results exhibited that restored miR-124 expression could lead to ceramide accumulation in tumor cells *via* inhibiting SphK1 expression.

### MiR-124-mediated apoptosis and regulation of pro-apoptotic and anti-apoptotic proteins

It is known that the interconversion of ceramide-sphingosine-S1P directs cells towards either an apoptotic program by ceramide and sphingosine or a survival program by S1P through interaction with various proteins, especially the Bcl-2 family. Therefore, we first analyzed whether JHU-22^miR124^ tumor xenografts had a higher frequency of apoptosis than JHU-22^vec^ xenografts using TUNEL assay. We then analyzed a set of key apoptosis-regulating proteins, especially the Bcl-2 family, in cultured cells and tumor xenografts using Western blotting and immunohistochemistry.

**Figure 8:**
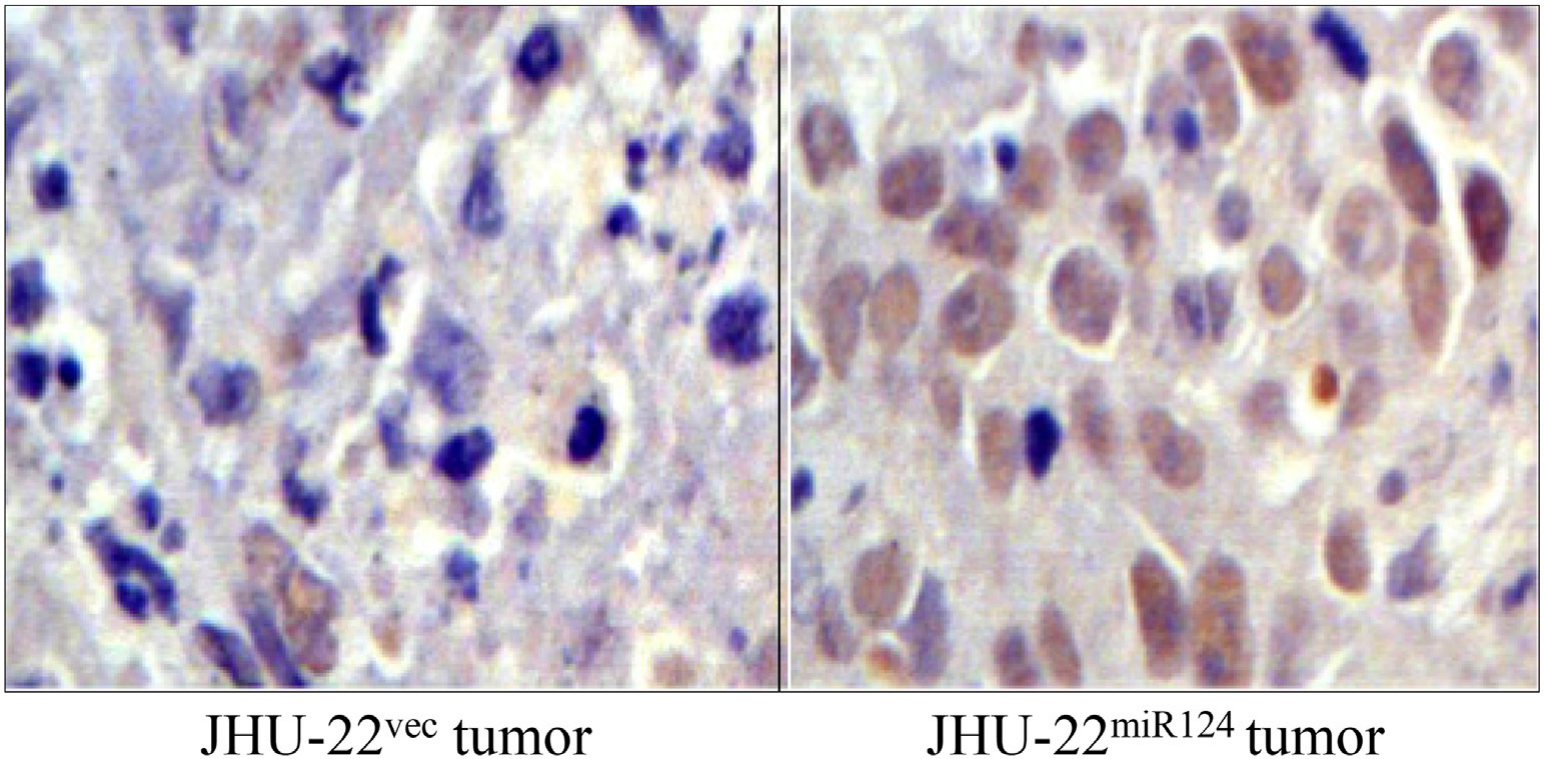
TUNEL assay showing a significantly higher number of apoptotic cells in JHU-22miR124 than in JHU-22vec tumor xenografts

**Figure 9:**
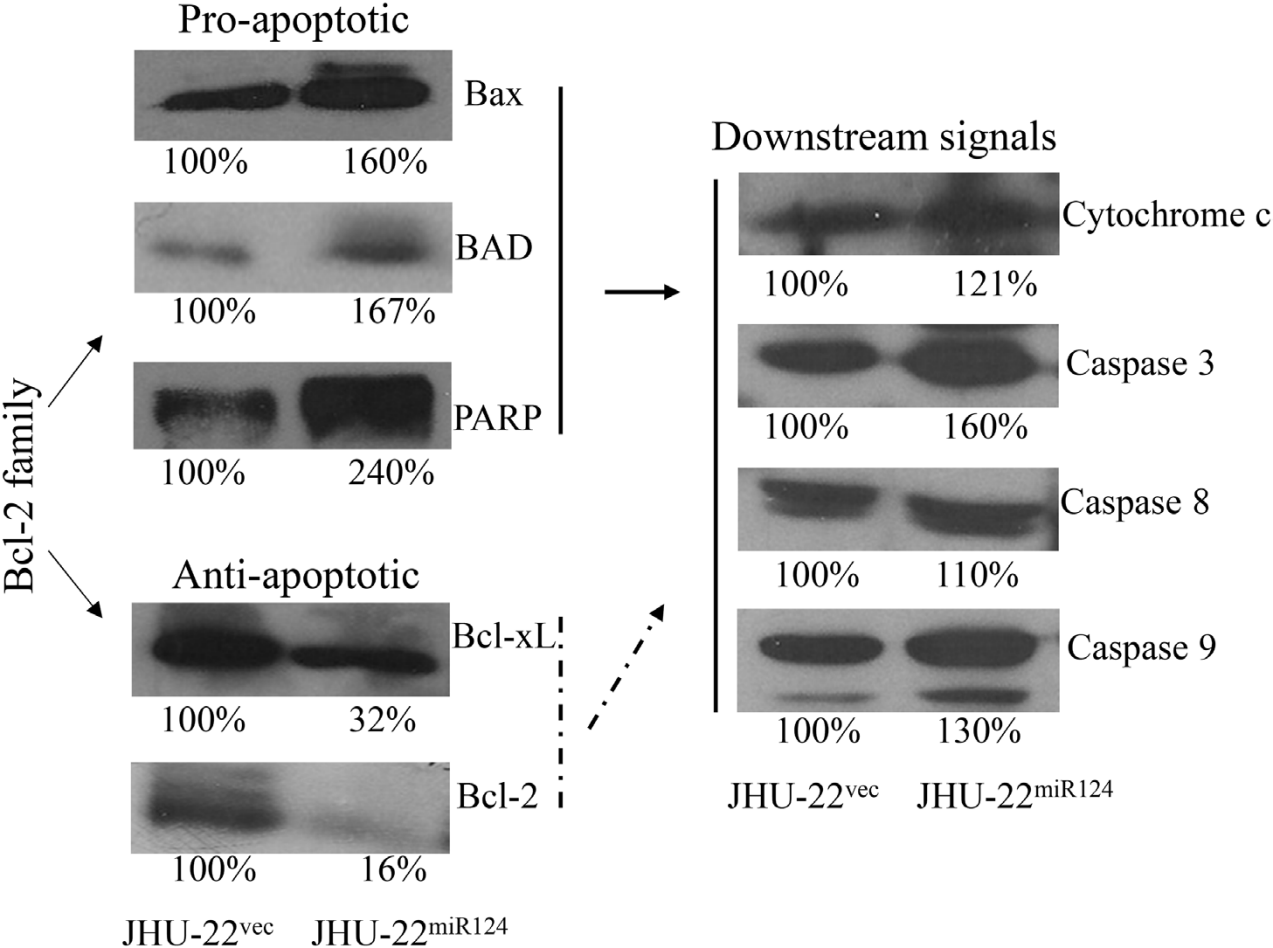
Western blot analysis of the expression of Bcl-2 family members and downstream signals, showing more than 60% higher expression for the pro-apoptotic members Bax, BAD and PARP; more than 60% lower expression for the anti-apoptotic members Bcl-2 and Bcl-xL; and 10% to 60% increased expression of the Bcl-2 family member downstream signals cytochrome c, caspase-3, caspase-8 and caspase-9 in JHU-22^miR124^ than in JHU-22^vec^ cells The percentage represents the expression of a protein in JHU-22^miR124^/JHU-22^vec^. β-actin was used for normalization. The specific band intensity was determined with the ImageJ software (NIH, Bethesda, MD). The gels used for Western blot analysis were 8% SDS-PAGE.

As shown in Figure 8, a significantly higher number of apoptotic cells was detected in the JHU-22^miR124^ xenografts than in the JHU-22^vec^ xenografts by TUNEL assay. Analysis of the Bcl-2 family members revealed that restored miR-124 expression resulted in significant changes in their expression levels. The pro-apoptotic members, including Bax, BAD and PARP, showed more than 60% higher expression levels in JHU-22^miR124^ than in JHU-22^vec^ cells (Figure 9). In contrast, the expression levels of anti-apoptotic members Bcl-2 and Bcl-xL decreased more than 60% in JHU-22^miR124^ cells (Figure 9).

**Figure 10:**
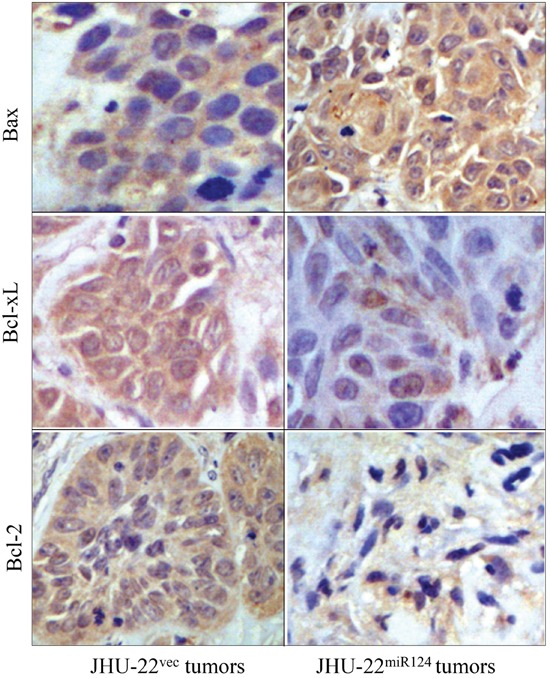
Immunohistochemical analysis of Bax, Bcl-xL and Bcl-2 expression in tumor xenografts, showing consistent expression patterns of these apoptosis-regulating proteins between cultured cells and tumor xenografts

The Bcl-2 family members are known to induce (pro-apoptotic members) or inhibit (anti-apoptotic members) the release of cytochrome c into the cytosol, which, once there, activates caspases that leads to cell apoptosis. We therefore further analyzed the level of cytochrome c, as well as the levels of caspase-3, caspase-8, and caspase-9. Western blotting demonstrated a higher level of cytochrome c in JHU-22^miR124^ cells than in JHU-22^vec^ cells (Figure 9). The expression levels of caspase-3, caspase-8 and caspase-9 also increased in JHU-22^miR124^ cells, varying from 10 to 60% increase (Figure 9). Immunohistochemical analysis with tumor xenografts verified the results of Western blotting with culture cells, showing similar expression patterns of Bax, Bcl-2, and Bcl-xL between culture cells and tumor xenografts (Figure 10).

## DISCUSSION

Growing evidence shows that miRNAs play a variety of crucial regulatory functions related to cell growth, development, and differentiation. These miRNAs repress gene expression and have been linked to various types of cancer [3, 7, 9]. However, target discovery of these cancer-associated miRNAs is still lagging, despite recent progress in emerging candidate targets. To identify those miRNAs that function abnormally in HNSCC, we first screened a group of cell proliferation-associated miRNAs for their expression profiles in human HNSCC. We found that the expression of miR-124 was significantly lower in tumors than in cancer-adjacent normal tissues, suggesting that miR-124 may act abnormally in HNSCC. To understand its role, we introduced miR-124 into cancer cells and restored its expression. Interestingly, restored expression of miR-124 led to significant inhibition of cell proliferation *in vitro* and of tumor xenograft growth in animals. The restored miR-124 expression also resulted in tumor cell arrest in the G1 phase of cell cycle, as well as in formation of smaller and fewer colonies. These results indicate that miR-124 acts as a tumor suppressor in HNSCC. Consistent to our findings, miR-124 has also been reported to be down-regulated and to act as a tumor suppresser in several other types of cancer [28–32]. The findings also prompted us to study its downstream signaling in HNSCC.

Literature search has revealed different targets of miR-124 in different tissues and tumor types, such as Foxq1 in nasopharyngeal cancer, CCAAT/enhancer-binding protein-α in macrophages, PTBP1 in neuronal differentiation, and CAV1 and FLOT1 in renal clear cell carcinoma [18–20]. In HNSCC, the epithelial-restricted with serine box/epidermal growth factor receptor (EGFR) and ITGB1 have been reported to be the targets of miR-124 [21, 33]. By a comprehensive search, we found that the 3′-UTR sequence of SphK1 mRNA carries a complementary site for the seed region of miR-124. Subsequent analysis revealed a close and negative relationship between miR-124 and SphK1 expression in both HNSCC cell lines and tumors. We hypothesized that SphK1 is a target of miR-124. To test our hypothesis, we constructed and co-transfected the 3′-UTR-Luc-SphK1 luciferase reporter vector with the miR-124 expression vector into HEK293T cells. The results demonstrated that SphK1 was a direct target of miR-124 in HNSCC, inhibiting the SphK1 expression by specifically binding with the complementary 3′-UTR sequence motif of SphK1.

In mammals, SphKs are an evolutionary conserved lipid kinase family with two isoforms, SphK1 and SphK2 [34, 35]. Although SphK1 and SphK2 appear to have opposing roles in promoting cell growth and apoptosis, respectively, they can also substitute for each other. SphK1 phosphorylates sphingosine to produce S1P, serving as a key regulator in response to the balance of ceramide-sphingosine-S1P rheostat, where the balance is critical in determining cell fate [36, 37]. Ceramide is associated with the anti-proliferative and cell death pathways, such as senescence and apoptosis. By contrast, S1P stimulates cell proliferation and survival pathways. Interestingly, SphK1 has been found to have high expression in many types of cancer and inhibition of its activity subsequently leads to accumulation of ceramide in tumor cells [38–40]. Consistent with these findings, we also found a higher level of ceramide accumulation and apoptosis in miR-124-expressing HNSCC cells *in vitro* and in tumor xenografts in animal models, suggesting a mechanism of miR-124-SphK1-Ceramide pathway in HNSCC.

**Figure 11:**
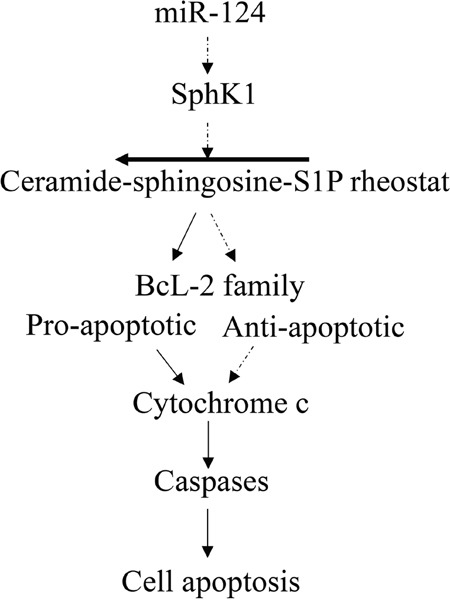
Schematic diagram of the miR-124 signaling pathway for regulating tumor cell apoptosis in HNSCC MiR-124 inhibits the expression of SphK1, which leads to the accumulation of ceramide in cells. Accumulated ceramide further activates pro-apoptotic members and inhibits anti-apoptotic members of the Bcl-2 family, which induces the release of cytochrome c into the cytosol. Once cytochrome c is in the cytosol, it activates caspases, leading to tumor cell apoptosis. Solid arrows indicate induction and dashed arrows indicate inhibition of the expression of downstream signals.

Ceramide accumulation in tumor cells has been commonly observed following treatment with various apoptotic agents [40, 41]. Because of its apoptosis-inducing effects in cancer cells, ceramide has been termed as “tumor suppressor lipid” [42, 43]. Although the specific role of ceramide in cell death and the mechanism by which this lipid regulates apoptosis remain elusive, there is evidence showing that Bcl-2 family members are involved in the ceramide-mediated apoptosis pathways [44, 45]. Bcl-2 is a family of evolutionarily related proteins which can be either pro-apoptotic or anti-apoptotic [46, 47]. Bcl-2 family proteins can induce (pro-apoptotic members) or inhibit (anti-apoptotic members) the release of cytochrome c into the cytosol, and subsequently regulate the expression of caspase proteins and apoptosis of tumor cells [48]. As expected, we detected a significantly higher number of apoptotic cells in JHU-22^miR124^ xenografts, compared with JHU-22^vec^ xenografts. Correspondingly, the pro-apoptotic members Bax, BAD and PARP increased more than 60% and the anti-apoptotic members Bcl-2 and Bcl-xL decreased more than 60% in their expression in JHU-22^miR124^ xenografts. This was accompanied by increased expression levels of cytochrome c, caspase-3, caspase-8, and caspase-9 in JHU-22^miR124^ cultured cells and tumor xenografts. We could conclude that the ceramide accumulation-mediated apoptosis signaling, together with the expression changes of Bcl-2 family members, is at least partially responsible for the inhibitory effects of miR-124 on HNSCC tumor growth (Figure 11).

In conclusion, we identified that miR-124 is down-expressed in HNSCC. Restored expression of miR-124 could effectively inhibit HNSCC cell proliferation in culture and tumor growth in mice, acting as a tumor suppressor. A mechanism underlying the suppressive effect of miR-124 could be outlined as a pathway of miR-124-SphK1-ceramide-Bcl-2 family-cytochrome c-caspases. In this pathway, miR-124 inhibits the SphK1 activity directly, which moves the ceramide-sphingosine-S1P rheostat toward accumulation of ceramide in tumor cells, and consequently, activates the pro-apoptotic members and inhibits the anti-apoptotic members of the Bcl-2 family. Ultimately, induced release of cytochrome c into the cytosol activates the caspase proteins, leading to cell apoptosis (Figure 11). As the next step, it will be interesting to understand whether the expression level of miR-124 is different in different stages of HNSCC and whether the altered expression of miR-124 is associated with the development of HNSCC resistance to chemotherapy, EGFR inhibition, and other therapies.

## MATERIALS AND METHODS

### Chemicals and antibodies

All chemicals used in the present study were of the highest grade from Sigma (St. Louis, MO). The miR-124 forward primer (5′- TAAGGCACGCGGTGAATGCC-3′) was synthesized at Sigma according to the miRBase Sequence database (Version 12.0). The antibody against SphK1 was obtained from Santa Cruz Biotechnology (Santa Cruz, CA), and those against Bax, Bad, Bcl-2, Bcl-xL, PARP, cytochrome c, Caspase-3, Caspase-8, Caspase-9, and β-actin were purchased from Sigma. The anti-ceramide antibody was purchased from ALEXIS Biochemicals (San Diego, CA).

### Human HNSCC tissues and cell lines

A total of ten HNSCC tumor tissues and five tumor-adjacent normal tissues were obtained from patients who underwent surgery during 2004-2005 at Johns Hopkins University. The protocol of sample collection and related studies were approved by the Institutional Review Board of the Johns Hopkins University. The HNSCC cell lines used in the present study included JHU-13, JHU-22, and JHU-29, which are originally established and characterized at Johns Hopkins University. The hTERT-transformed noncancerous human oral keratinocyte cell line (OKF-6) was a generous gift from Dr. James Rheinwald at Harvard University. All HNSCC cell lines were routinely cultured in RPMI1640 medium fortified with 10% fetal bovine serum and antibiotics. The OKF-6 cells were cultured in serum-free keratinocyte medium (Thermo Fisher Scientific, Waltham, MA). All experiments were performed with the cells in the logarithmic phase of growth.

### RNA isolation and QRT-PCR

Total RNA was isolated from frozen tissues and from cultured cells with the Trizol RNA isolation reagent (Invitrogen) and RNeasy Mini Kit (Qiagen, Germantown, MD), respectively, following the manufacturers’ protocols. Following polyadenylation of the RNA with polyA polymerase, the first-strand cDNA was synthesized and the QRT-PCR was conducted using the High-Specificity miRNA Detection Kit (Stratagene, La Jolla, CA). Three controls were included as no RNA template control, no polyA polymerase control, and an endogenous control. The miRNA expression relative to the U6 RNA expression was determined using the ΔCt method. The fold-change for miRNA expression level was calculated with 2^−ΔΔCt^.

### Construction of EGFP-miR-124 expression and control vectors, and generation of the miR-124-expressing JHU-22^miR124^ subline and the control JHU-22^vec^ subline

The EGFP-miR-124 expression vector was constructed based on a lentiviral expression system [49]. We first modified the commercial pLVX-Tight-Puro vector (Clontech, Mountain View, CA) by replacing the P_tight_ promoter with an expression cassette containing P_CMV_ promoter, EGFP, miRNA linker, and pre-miR-124. The pre-miR-124 double-strand sequence was synthesized based on the miRBase Sequence database (Version 12.0). Every step of the vector construction was verified by DNA sequencing. A mock EGFP vector without the pre-miR-124 sequence was also constructed as a control. The lentivirus particles containing the EGFP-miR-124 vector or the EGFP control vector were produced using the lentiphos^TM^ HT packaging system (Clontech) following the manufacturer's instruction.

To establish the miR-124-expressing sublines, JHU-22 cells were pre-seeded in the 6-well plates overnight, infected with 200 μl of lentivirus containing the EGFP-miR-124 vector or the EGFP control vector for 2 h, and then added 2 ml of RPMI1640 medium with 10% FBS to each well. After 48 h, infected cells were selected with fresh medium containing 5 ng/ml puromycin for 4-5 passages. Those cells with stably expressing either EGFP-miR-124 (JHU-22^miR124^) or EGFP alone (JHU-22^vec^) were monitored by EGFP expression under fluorescence microscopy and clones with the highest expression of miR-124 were selected with QRT-PCR.

### Construction of 3′-UTR-Luc-SphK1 and mutated 3′-UTR-Luc-SphK1 luciferase reporter vectors and luciferase assay

The 3′-UTR-Luc-SphK1 vector and the mutated 3′-UTR-Luc-SphK1 vector were constructed by inserting a fragment of the 3′-UTR SphK1 mRNA and a mutated 3′-UTR SphK1 mRNA into the phCMV-FSR luciferase reporter vector (Genlantis, San Diego, CA), respectively. The 3′-UTR-Luc-SphK1 vector carried a putative miR-124 complementary binding site (position 16–22 of SphK1 3′-UTR). The 3′-UTR-Luc-SphK1 vector or the mutated 3′-UTR-Luc-SphK1 vector were co-transfected, specifically, with either the EGFP-miR-124 expression vector or the mock EGFP-vector into the HEK293T cells (ATCC, Manassas, VA) using calcium phosphate. The luciferase activity was quantified 48 h after transfection using the Xenogen IVIS bioluminescence imaging system (PerkinElmer, Waltham, MA).

### MTT (3-(4, 5-Dimethylthiazol-2-yl)-2, 5-diphenyltetrazolium bromide) and colony formation assays

MTT assay was used to estimate cell viability and colony formation assay was used to determine colon-forming capability. For MTT assay, cells were seeded into 96-well plates and incubated for various times. MTT solution (Sigma, St Louis, MO) was then added to cells and further incubated for 4 h, followed by addition of dimethyl sulfoxide solution for 30 min at room temperature. The absorbance at 560 nm was measured spectrophotometrically (Microplate reader, Bio-Rad, Hercules, CA). For the colony formation assay, 500 cells of JHU-22^vec^ or JHU-22^miR124^ per well were seeded into the BD Falcon 6-well plates (Palo Alto, CA). After nine days, colonies were stained with 0.1% trypan blue in 50% ethanol. Those colonies containing >50 cells were considered to be a viable clonogenic cell and counted. At least two independent experiments were performed with triplicate samples.

### Flow cytometry

Flow cytometry was conducted for cell cycle analysis and determination of protein expression levels. For cell cycle analysis, cells were first pre-cultured without serum overnight and then cultured with serum for additional 24 h. The cells were fixed with 80% cold ethanol, treated with 1 mg/ml RNase A for 30 min at room temperature, and then incubated for 30 min in the dark with 0.5 ml of 50 mg/ml propidium iodide. The distribution of cells throughout the cell cycle was analyzed by FACStar flow cytometry (Becton Dickinson & Co., San Jose, CA). To determine protein expression levels, cells were collected and fixed with 4% paraformaldehyde. The fixed cells were washed with PBS and incubated with the defined primary antibodies for 2 h at room temperature. After thorough washing, cells were incubated with R-phycoerythrin-conjugated anti-rabbit antibody for 40 min at room temperature. Those cells with defined protein expression were determined by FACStar flow cytometry.

### Growth of JHU-22^miR124^ and JHU-22^vec^ tumor xenografts in athymic nude mice

Four-week-old male Balb/c athymic nude mice (Nu/Nu) were obtained from Harlan Sprague Dawley, Inc. (Indianapolis, IN). Mice were housed in a temperature-controlled room (74 ± 2 °F) with a 12-h alternating light-dark cycle. The JHU-22^vec^ and JHU-22^miR124^ cells (1×10^6^) were inoculated into the subcutaneous tissue of the left and right lower back of each mouse, respectively (*n* = 8). The mice were housed for 4 weeks and then euthanized. The tumor tissues were removed and processed for RNA and protein extraction and for formalin fixation for pathological studies. During the experiments, tumor volume and mouse body weight were measured every 3-4 days. The research protocol was approved by the Howard University Animal Care and Use Committee.

### Western blot analysis and immunohistochemistry

Whole-cell proteins were extracted from the JHU-22^vec^ and JHU-22^miR124^ cells and tumor xenografts using RIPA lysis buffer (Santa Cruz Biotechnology). Thirty μg of total proteins were separated on the 8% SDS-PAGE gel and then transferred to the polyvinylidene difluoride membrane. The membranes were probed sequentially with primary antibodies and secondary horseradish peroxidase-conjugated antibodies, and developed with the ECL detection system (Bio-Rad, Hercules, CA). The protein to β-actin expression ratio was determined with the ImageJ software (NIH, Bethesda, MD). For immunohistochemistry, LSAB2 System-HRP Kit (DakoCytomation, Carpinteria, CA) was used. Deparaffinized tissue sections were first treated with 3% hydrogen peroxide, and then incubated with primary antibodies. The specific signals were developed with streptavidin reagent and DAB Substrate (Abcam, Cambridge, MA).

### Mass spectrometry analysis

Identification and quantification of ceramide species were performed by shotgun lipidomics as previously described [50, 51]. Briefly, cells grown in 10-cm culture dishes were harvested, washed, and centrifuged. The pellets were homogenized in 0.5 mL of 20 mM LiCl with a glass tissue grinder at 0 °C. Lipids were extracted from each homogenate utilizing 50 mM LiOH in the aqueous layer in the presence of N17:0 Cer (2 nmol/mg of protein). The final lipid residue was resuspended in 0.2 mL of 1:1 chloroform/methanol. ESI mass spectral analyses of ceramides were performed utilizing a Finnigan TSQ-7000 spectrometer equipped with an electrospray ion source. Lipid extract samples were directly infused into the ESI chamber using a syringe pump at a flow rate of 1 μL/min. Prior to the ceramide analyses of lipid extracts from biological samples, LiOH in methanol (50 nmol/mg of protein) was added. Ceramide molecular species were directly quantitated by comparisons of ion peak intensities with that of internal standard (N17:0 Cer) after correction for ^13^C isotope effects. Quantitative data were normalized to the protein content of the samples and all data are presented as the mean ± SE of minimal six independent preparations.

### Terminal deoxynucleotidyl transferase-mediated dUTP nick end labeling (TUNEL) assay

Apoptotic cells in deparaffinized tissue sections were determined using TUNEL assay kit (GeneTex, Irvine, CA) following the manufacturer's protocol. The data were semi-quantified based on the positive cell number and staining density under microscopy.

### Statistical analysis

The quantitative data were presented as the mean ± S.D. T-test was used to determine the statistical significance. Significant difference was considered at *P*<0.05.
